# Highlighting the Need for Systems-Level Experimental Characterization of Plant Metabolic Enzymes

**DOI:** 10.3389/fpls.2016.01127

**Published:** 2016-07-28

**Authors:** Martin K. M. Engqvist

**Affiliations:** Independent ScholarGhent, Belgium

**Keywords:** organic acid metabolism, functional gene annotation, high-throughput platforms, bioinformatic predictions, proteome sequence homology, crop species, forestry trees, enzyme biochemical characterization

## Abstract

The biology of living organisms is determined by the action and interaction of a large number of individual gene products, each with specific functions. Discovering and annotating the function of gene products is key to our understanding of these organisms. Controlled experiments and bioinformatic predictions both contribute to functional gene annotation. For most species it is difficult to gain an overview of what portion of gene annotations are based on experiments and what portion represent predictions. Here, I survey the current state of experimental knowledge of enzymes and metabolism in *Arabidopsis thaliana* as well as eleven economically important crops and forestry trees – with a particular focus on reactions involving organic acids in central metabolism. I illustrate the limited availability of experimental data for functional annotation of enzymes in most of these species. Many enzymes involved in metabolism of citrate, malate, fumarate, lactate, and glycolate in crops and forestry trees have not been characterized. Furthermore, enzymes involved in key biosynthetic pathways which shape important traits in crops and forestry trees have not been characterized. I argue for the development of novel high-throughput platforms with which limited functional characterization of gene products can be performed quickly and relatively cheaply. I refer to this approach as systems-level experimental characterization. The data collected from such platforms would form a layer intermediate between bioinformatic gene function predictions and in-depth experimental studies of these functions. Such a data layer would greatly aid in the pursuit of understanding a multiplicity of biological processes in living organisms.

## Two Complementary Approaches to Studying Living Organisms

The reductionist approach toward studying living organisms involve investigating single components in isolation. This approach has been greatly successful at explaining a multiplicity of biological processes. A large body of experimental data such as gene sequences, gene expression patterns and gene product properties have been collected and deposited into public databases. The systems approach toward studying living systems focuses on modeling and studying the interplay between a large number of single components, as well as properties emerging from that interplay. It therefore builds on – and is complementary to – the reductionist approach.

Both the reductionist as well as the systems approaches have strengths and weaknesses. The reductionist approach generates in-depth data, but is slow and may miss the bigger picture. Due to the large investment of time and resources required for this approach, even for characterizing the function of a single gene product, it cannot possibly be used to keep up with, or catch up to, the ever-increasing flood of genomic data. On the other hand, the systems approach is often reliant on bioinformatic annotation pipelines to automatically generate gene function predictions (Mueller et al., 2003; Tatusova et al., 2007; Zhang et al., 2010; Sucaet et al., 2012; Van Bel et al., 2012; Seaver et al., 2014). This leaves the systems-level approach vulnerable to false gene function predictions. This problem is compounded as an ever-increasing number of genomes are sequenced from organisms that are evolutionarily distant to well-characterized plant, animal, fungal and microbial model systems. With hundreds of plant genomes being available within the near future (Reddy et al., 2015) it is highly relevant to assess our current level of knowledge regarding gene functions in plants. However, gaining an overview of which functional annotations are supported by experimental evidence, and which are not, is often not straight-forward.

## The Diversity of Chemical Reactions Characterized in Crops and Forestry Trees is Low

To assess the state of experimental knowledge in plant species I focus on enzymes and use these to infer a general trend. Enzyme activities are classified using Enzyme Commission numbers (EC numbers), with each number indicating a specific type of reaction and the substrate(s), product(s) and co-factor(s) involved. This classification system enables rapid identification of functionally equivalent enzymes in different species. The BRENDA database^1^ is the main collection of enzyme functional data available to the scientific community (Schomburg et al., 2002; Chang et al., 2015). This resource (release from January, 6th 2016) was used to get an overview of the total number of unique EC numbers experimentally characterized for each of a set of 12 plant species. The EC numbers represent the diversity of chemical reactions known in these organisms. It is important to note that there is typically a delay from the publishing of a primary research article until the enzyme data can be retrieved from the BRENDA database. Data from enzymes characterized more recently may therefore not be included in this analysis. *Arabidopsis thaliana* (arabidopsis) was included in the analysis due to its long-standing role as a plant model species. *Zea mays* (maize), *Oryza sativa* (rice), *Triticum aestivum* (wheat), *Solanum tuberosum* (potato), *Manihot esculenta* (cassava) and *Glycine max* (soybean) were included as they are the six most important crop species by annual production globally^2^. *Picea abies* (Norway spruce), *P. glauca* (white spruce), *P. sitchensis* (sitka spruce), *Pinus taeda* (loblolly pine), and *Populus trichocarpa* (poplar) were also included, representing five economically important forestry tree species. Data from *Homo sapiens* (humans), *Saccharomyces cerevisiae* (baker’s yeast), and *Escherichia coli* are included to put the plant data into context.

**FIGURE 1:**
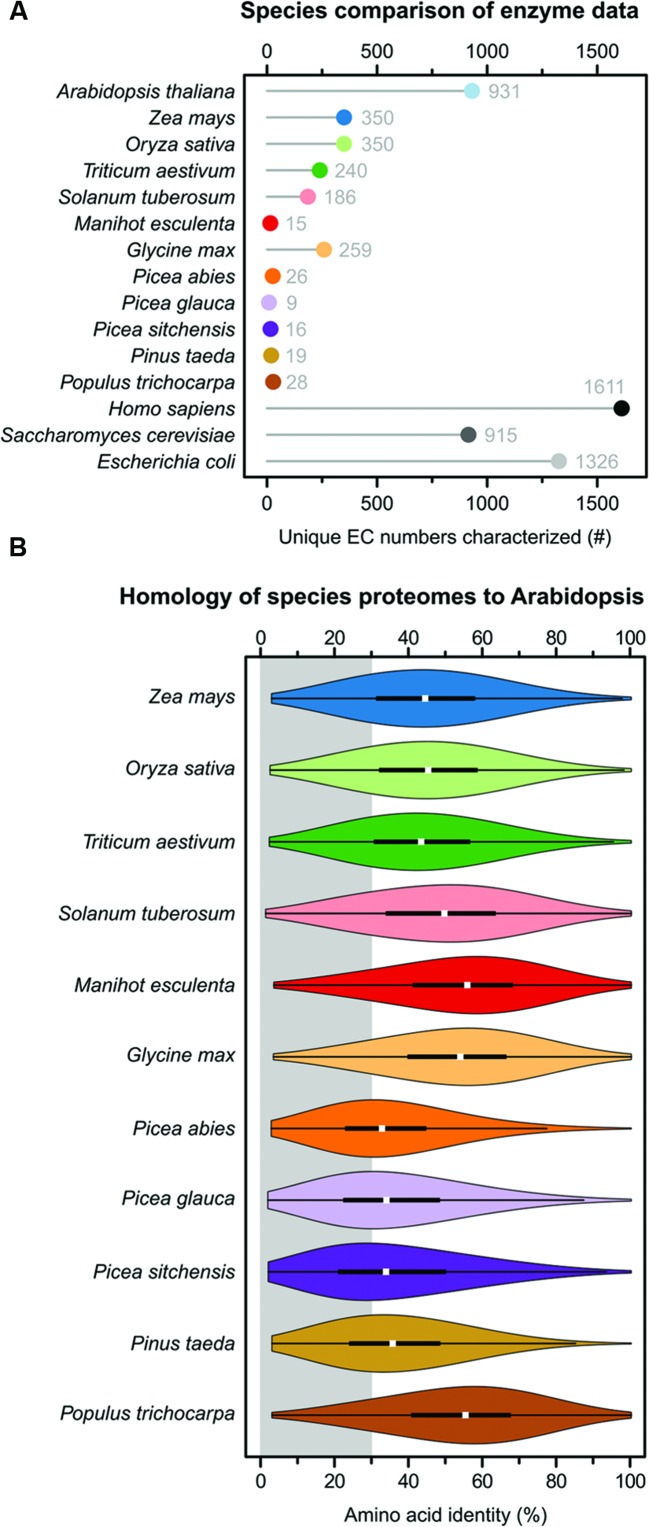
**Species comparison of available biochemical data and proteome homology. (A)** A Cleveland dot plot showing how many unique EC numbers exist for each species in the BRENDA database. Each EC number classifies a specific catalyzed reaction, the numbers thus indicate the diversity of chemical reactions known from the different species. **(B)** A violin plot of amino acid identity of the arabidopsis proteome compared to the proteomes of eleven plant species. Each “violin” in the plot represents ∼28,000 pairwise comparisons with amino acid identities calculated based on global sequence alignments. The white dot represents the median. The broad black bar represents the upper and lower quartile, which contain 50% of the data points. The thin black lines represent the upper and lower adjacent values. The outer plot shape is a kernel density plot that visualizes the probability distribution of the data. The gray area ranges from 0 to 30% identity and indicates the “twilight zone” of protein–protein comparisons where proteins similar in sequence no longer perform the same function. Scientific to common name translations: *A. thaliana* – arabidopsis, *Z. mays* – maize, *O. sativa* – rice, *T. aestivum* – wheat, *S. tuberosum* – potato, *M. esculenta* – cassava, *G. max* – soybean, *P. abies* – Norway spruce, *P. glauca* – white spruce, *P. sitchensis* – sitka spruce, *P. taeda* – loblolly pine, and *P. trichocarpa* – poplar.

Unsurprisingly, the model species arabidopsis has the highest total of characterized EC numbers of all analyzed plants, with 931 (**Figure 1A**). This is similar in magnitude to the 915 EC numbers known from baker’s yeast, but much less than the 1,326 EC numbers known from *E. coli* and the 1,611 known from humans. It is striking that from this well-studied plant there are 395 (30%) fewer characterized EC numbers than from the bacterium *E. coli*. Maize, rice, wheat, potato and soybean range from 186 to 350 characterized EC numbers. Maize and rice have 350 characterized EC numbers, the highest total EC numbers of the crop species. This is 976 (73%) fewer characterized EC numbers than *E. coli*. Of the six analyzed crop species cassava has the fewest characterized EC numbers, with a grand total of 15 (**Figure 1A**). For all five tree species, similarly few EC numbers have been characterized as in cassava, ranging from 9 to 28 (**Figure 1A**). The diversity of chemical reactions which are experimentally characterized in the five tree species and cassava each represent less than 2.1% of those from *E. coli* and 1.7% from humans. When combining all unique, non-overlapping, EC numbers characterized in the 12 plant species the grand total is 1,240 – which is still less than the 1,326 EC numbers known from *E. coli* alone.

The small number of experimentally characterized enzymes in these economically important species means that the genes are instead either annotated based on bioinformatic predictions or remain functionally un-annotated. For some of these enzymes there may be other types of experimental data supporting their function, for example studies involving loss-of-function mutants or chemical genomics approaches, but that number is expected to be low. Un-annotated or miss-annotated genes diminish our capability to accurately model biological processes and studying emergent properties. This is especially true for systems biology approaches such as Gene Ontology (GO-term) enrichment analysis and genome-scale metabolic modeling approaches.

## Sequence Homology is Low between the Arabidopsis Proteome and those from Crops and Forestry Trees

Since enzyme functional annotations in plants are largely based on bioinformatic predictions it is relevant to ask how homologous the sequences used for these annotations are. Arabidopsis has the largest number of characterized EC numbers and enzyme annotation in crop species and forestry trees are thus likely based on arabidopsis homologs. I compared the sequence homology of the arabidopsis proteome with those of the six crop species and five tree species. Each arabidopsis protein longer than 120 amino acids was used in a protein-protein BLAST (Altschul et al., 1990) to identify the most homologous proteins in each of the proteomes. The results were filtered to retain only query-hit pairs where the alignable region was more than 70% of the length of the query. A global sequence alignment was generated for each query-hit pair using MUSCLE (Edgar, 2004). The percentage amino acid identity for each pair was then calculated on the basis of the alignments.

The amino acid identity scores for the entire proteomes were visualized as a violin plots using custom R^3^ scripts (**Figure 1B**). The portion of sequences below 30% identity – indicated with a gray background in the figure – is important since this represents the “twilight zone” of amino acid identity, where proteins similar in sequence no longer have the same overall fold or no longer perform the same function (Sander and Schneider, 1991; Rost, 1999; Konstantinidis and Tiedje, 2005). The proteomes of the monocotyledonous species maize, rice and wheat have median protein–protein identities of 43–45% compared with arabidopsis. 22–24% of the arabidopsis proteins have less than 30% amino acid identity to the proteins in these species and are thus in the “twilight zone” (**Figure 1B**). The proteomes of the dicotyledonous species potato, cassava, soybean and poplar have median identities of 49–56% compared with arabidopsis. 13–20% of the arabidopsis proteins have less than 30% amino acid identity to the proteins in these species (**Figure 1B**). The four gymnosperms Norway spruce, white spruce, sitka spruce and loblolly pine have by far the least homologous proteomes compared with arabidopsis, with median identities of 33–35%. A full 38–44% of the arabidopsis proteins have less than 30% amino acid identity to the proteins in these species and cannot be said to be functionally equivalent (**Figure 1B**).

The fact that large proportions of the proteomes share less than 30% identity with arabidopsis (**Figure 1B**), while at the same time little experimental data being available to support enzyme annotations (**Figure 1A**), indicates that there is an over-reliance on bioinformatic predictions in all of the analyzed species. This may lead to propagation of inconsistent or incorrect annotations among genomes. Furthermore, the reliance on bioinformatics also represent missed opportunities to discover unique properties of plant enzymes – and how those unique properties shape metabolism. Pathways and reactions which are unique to certain species also remain undiscovered.

To further highlight this problem I scoped the total number unique EC numbers annotated in the genomes – irrespective of whether the source of that annotation was experimental data or bioinformatic predictions. I downloaded the GO-term annotations for *E. coli*, baker’s yeast, and humans from the website of the Gene Ontology Consortium (Ashburner et al., 2000; Consortium, 2015)^4^ and annotations for arabidopsis, maize, rice (Indica variety), potato, cassava, soy and poplar from the Plaza 3.0 website (Proost et al., 2015)^5^. A source for GO-term annotations for wheat and the four gymnosperms could not be identified. The total number of unique GO-terms representing enzyme activities were: *E. coli* (1,201), baker’s yeast (1,029), humans (1,549), arabidopsis (1,354), maize (956), rice (1,058), potato (1,203), cassava (1,202), soy (1,222) and poplar (1,232). These numbers underscore the problems with the reliance on bioinformatic predictions. The dicotyledonous plants have a similar number of predicted EC numbers as arabidopsis, probably because their proteomes are similar. The proteomes of maize and rice are less similar to arabidopsis and also has fewer genes annotated with EC numbers. The fact that maize and rice have many fewer annotated EC numbers than *E. coli* and arabidopsis is likely explained by limitations in our ability to predict more, and not a true reflection of the actual EC number count in these plants. Once GO-term annotations for the four gymnosperms become available I expect those to contain far fewer predicted EC numbers than maize and rice, due to their low sequence similarity to the arabidopsis proteome.

## Many Enzymes Involved in Metabolism of Citrate, Malate, Fumarate, Lactate, and Glycolate in Crops and Forestry Trees have not been Characterized

**FIGURE 2:**
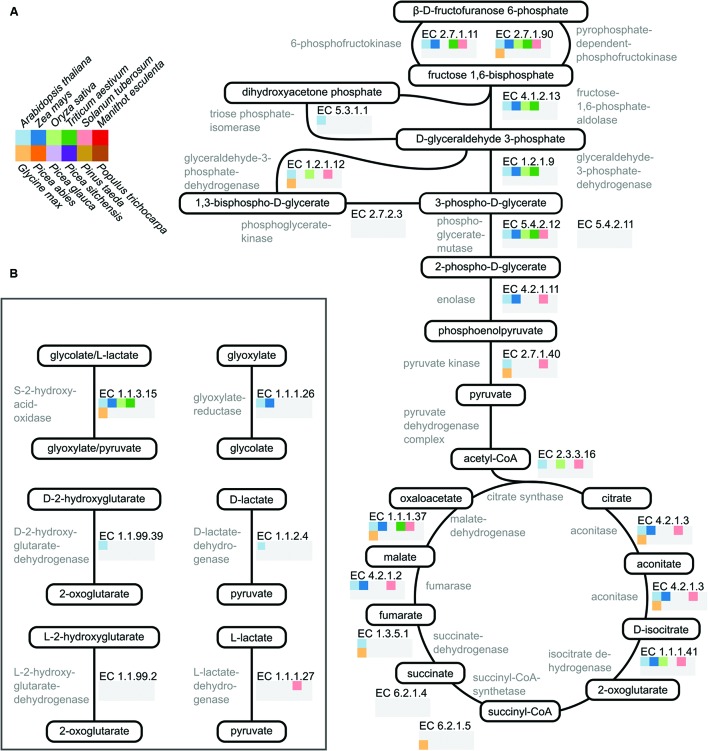
**Visualization of available biochemical data on pathways in plant core metabolism. (A)** A cartoon representation of the reactions comprising glycolysis and the TCA cycle. Metabolite names are encircled and shown in black. Enzyme names are shown in gray and their corresponding EC number shown in black. The array of squares below each EC number indicates whether data is available for that enzyme in the BRENDA database. For each species where data is present the square is shown in color according to the species key shown on the top left. If no data is present the square is colored gray. **(B)** A cartoon representation of six reactions involved in metabolism of 2-hydroxy acids. Coloring is identical to that in **(A)**. Scientific to common name translations: *A. thaliana* – arabidopsis, *Z. mays* – maize, *O. sativa* – rice, *T. aestivum* – wheat, *S. tuberosum* – potato, *M. esculenta* – cassava, *G. max* – soybean, *P. abies* – Norway spruce, *P. glauca* – white spruce, *P. sitchensis* – sitka spruce, *P. taeda* – loblolly pine, and *P. trichocarpa* – poplar.

The sum of EC numbers characterized for each species (**Figure 1A**) does not indicate which specific reactions, from which pathways, have been characterized. To look at a few pathways in detail I selected 20 reactions involved in glycolysis and the TCA cycle (**Figure 2A**). These reactions were chosen due to their central role in the metabolism of important organic acids such as citrate, malate, and fumarate. I also chose six reactions involved in metabolism of the 2-hydroxy acids glycolate, lactate, and hydroxyglutarate (**Figure 2B**), which are closely connected with central metabolism (Maurino and Engqvist, 2015). For each of the twelve plant species, and for each of the selected reactions, the presence or absence of data in the BRENDA database was visualized (**Figures 2A,B**).

In glycolysis and the TCA cycle, enzymes from arabidopsis, maize, and potato have been characterized for the majority – but not all – of the reactions (**Figure 2A**). Characterized enzymes from rice and wheat mainly participate in glycolysis whereas most of the characterized soybean enzymes participate in the TCA cycle. In the set of reactions studied here there is not a single enzyme characterized from cassava or any of the five tree species (**Figure 2A**). None of the 12 plant species analyzed had had all reactions from glycolysis and the TCA cycle characterized. For the six reactions involved in 2-hydroxy acid metabolism most of the twelve analyzed plant species had no characterized enzymes (**Figure 2B**). Arabidopsis represents an exception with enzymes characterized for four of the six EC numbers (**Figure 2B**). From maize, rice and wheat S-2-hydroxy acid oxidase (EC 1.1.3.15) has been characterized. Additionally, there is data for glyoxylate reductase (EC 1.1.1.26) in maize and for L-lactate dehydrogenase (EC 1.1.1.27) in potato (**Figure 2B**). It is important to note that some of these enzymes may have been characterized, but the information has yet to be included in the BRENDA database.

This analysis shows that for key metabolic reactions involved in organic acid metabolism there is little biochemical data available for most of the analyzed plant species. We therefore have a limited insight into species-specific features of important biological processes involving these organic acids, such as central metabolism, C4 metabolism, transient carbon storage, reductive energy cycling between subcellular compartments, and stomatal function. Furthermore, there are likely enzymes important for these processes which are currently miss-annotated or un-annotated, in particular in plant species which are evolutionarily distant to arabidopsis. Our ability to accurately model and understand these processes is thus diminished, with negative consequences for our capability to generate high-yielding, robust crops and forestry trees to meet a changing climate.

## Most Enzymes Involved in Metabolic Pathways which Shape Key Traits in Crops and Forestry Trees have not been Characterized

To gain a broader view of which parts of metabolism have been characterized, and which have not, custom Python^6^ scripts were used to map the EC numbers characterized from arabidopsis and the six crop species to the Kyoto Encyclopedia of Genes and Genomes (KEGG) global metabolism overview map (Kanehisa and Goto, 2000; Kanehisa et al., 2016). The resulting maps cannot be faithfully reproduced in the body of this text due to their large size and high detail, they are instead included in the Supplementary Figures S1–S7. Enzymes characterized from arabidopsis cover many parts of the metabolic pathways in the KEGG overview map (Supplementary Figure S1). Extensive gaps, where no enzymes have been characterized, exist in nucleotide metabolism, amino acid metabolism, carotenoid biosynthesis, glycan biosynthesis, as well as in metabolism of cofactors and vitamins (Supplementary Figure S1). For maize, rice, wheat, potato and soybean, enzymes in central metabolism, starch biosynthesis, amino acid biosynthesis, nucleotide metabolism and glycerosphingolipid metabolism are more numerous than in other parts of metabolism (Supplementary Figures S2–S5 and S7). Even so, there are significant gaps in many of these pathways. In cassava, the handful of characterized enzymes participate mostly in cyanoamino acid metabolism and starch metabolism (Supplementary Figure S6). Critically, pathways for biosynthesis of fatty acids, lipids, starch, amino acids as well as co-factors and vitamins in crop plants – all of which are critical for biosynthesizing edible biomass and determining its nutritional value – are not complete for any of the analyzed species. Our understanding of the molecular underpinnings of important traits such as yield, nutritional value, biomass composition, abiotic stress tolerance as well as disease and herbivore resistance in crop species and economically important forestry trees is severely limited by not having experimental data for many of the enzymes participating in these pathways.

## Conclusion

In this perspective article I focus on enzymes, but the state of knowledge is expected to be similarly weak, or even worse, for other types of gene products, such as transcription factors, non-coding RNA molecules (miRNA, siRNA, snRNA, etc.), and structural proteins in these species. Our understanding of the molecular underpinnings of important traits such as yield, nutritional value, biomass composition, abiotic stress tolerance as well as disease and herbivore resistance in crop species is thus severely limited. This limitation is two-fold. First, when using omics approaches in crop plants to compile lists of genes which may be important for specific crop traits, one is often left guessing which functions the gene products perform. This is true for enzymes as well as other types of gene products. Second, if one wishes to up- or down-regulate specific metabolic pathways to improve crop traits, one rarely knows with certainty which enzymes or regulatory proteins to target. Having experimental data available for the majority of gene products in economically important plant species is pivotal for a deep molecular understanding of plants, for our ability to model plant systems, and for improving plants through biotechnology. A powerful reminder of how much we have left to learn about living organisms comes from the synthetic minimal bacterial cell JCV-syn3.0, described earlier this year (Hutchison et al., 2016). From the microbe’s 473 genes, all of which are essential for robust growth, 149 (32%) are of unknown function.

## Perspectives

The path forward to generating the quantities of data needed for accurate annotation of the stream of incoming genomes does not lie in intensified efforts for in-depth functional characterization of gene products. Such studies are incredibly valuable, and must certainly be continued, but are slow and cannot be sufficiently scaled up to meet the flood of new plant genomic data. Instead we need to develop novel high-throughput platforms for performing “systems-level experimental characterization” – rapid and limited functional characterization of most genes in a genome. Combining these types of platforms with *in vivo* experiments, such as flux balance analysis, should serve to rapidly expand our knowledge of living organisms. Different types of platforms need to be established for the characterization of transcription factors, enzymes and non-coding RNAs. The data collected by each platform would be limited in scope. For example, one platform might perform high-throughput determination of the substrate scope and specificity of enzymes, but none of the other enzyme properties. Another platform might determine the subcellular localization of proteins in high throughput, but no +other properties relating to these proteins. The resulting data would form an intermediate layer between bioinformatic predictions and in-depth functional characterization of gene products. This data layer would strengthen the foundation of systems-biology approaches, provide a starting point for in-depth gene-function studies, and pave the way for more accurate bioinformatic predictions for genes in newly sequenced plant genomes.

A few platforms and technologies that fulfill this purpose do exist. One example is the transcriptome revolution brought on by RNAseq, with which one can relatively cheaply determine the expression levels of the majority of genes in a genome (Edwards et al., 2013; McCormack et al., 2013; Mutz et al., 2013). Another example is chemical genomics, which, in combination with genetics, also hold great promise of generating much needed functional data for a large number of genes (Barglow and Cravatt, 2007; Robert et al., 2009; McCourt and Desveaux, 2010). Yet another example is a platform leveraging CRISPR/Cas9 and serine integrases for creating protein fusions *in vivo* and using these to investigate protein–protein interactions and subcellular localization of gene products (Mulholland et al., 2015).

A platform for systems-level characterization plant enzymes can be achieved with existing technologies through combining three types of optimization: (i) using efficient high-throughput experimental methods, (ii) seeking to obtain only information on the kinetic constants and substrate scope for each enzyme under standardized conditions and (iii) testing homologous enzymes from numerous species in the same assay at the same time. The combination of these approaches would allow a single researcher to generate data on several hundred, or with automation, thousand enzymes per year. The required high-throughput methods are well-established and used routinely in the field of directed evolution. These methods enable efficient cloning, expression, quantification, and measurement of a large number of enzyme variants in a single assay. In directed evolution enzyme libraries are generated by the researcher in a laboratory – were each enzyme in the library typically differ by no more than a handful of mutations. The key insight is to realize that nature has generated the equivalent of enzyme libraries through the process of speciation. For example, all citrate synthases from a set of 20–30 plant species is analogous to a small enzyme library and can be tested in a single assay to determine their kinetic constants. It will be important to test each set of enzymes with a panel of substrates as to not only test the bioinformatic prediction, but also possible side activities or alternate main activities. It should be possible to further expand this approach to testing entire protein families with a small set of carefully chosen enzyme assays in a single experiment. Access to high-quality gene sequence information from plant genomes, as well as low gene synthesis costs, are key requisites for this approach.

## Author Contributions

ME was solely responsible for conceiving and executing this study as well as for authoring this manuscript.

## Conflict of Interest Statement

The author declares that the research was conducted in the absence of any commercial or financial relationships that could be construed as a potential conflict of interest. The reviewer WA declared a past co-authorship with the author to the handling Editor, who ensured that the process met the standards of a fair and objective review.

## Footnotes

1 http://www.brenda-enzymes.org/

2 http://faostat.fao.org/

3 http://www.r-project.org/

4 http://geneontology.org/

5 http://bioinformatics.psb.ugent.be/plaza/

6 http://www.python.org/
