# Supplementary material for: Highlighting the Need for Systems-Level Experimental Characterization of Plant Metabolic Enzymes
Source: Front Plant Sci. 2016 Jul 28;7:1127. doi: 10.3389/fpls.2016.01127 (PMC4963410; doi:10.3389/fpls.2016.01127)
Supplement: Supplementary file 5 [file Image_5.PDF]

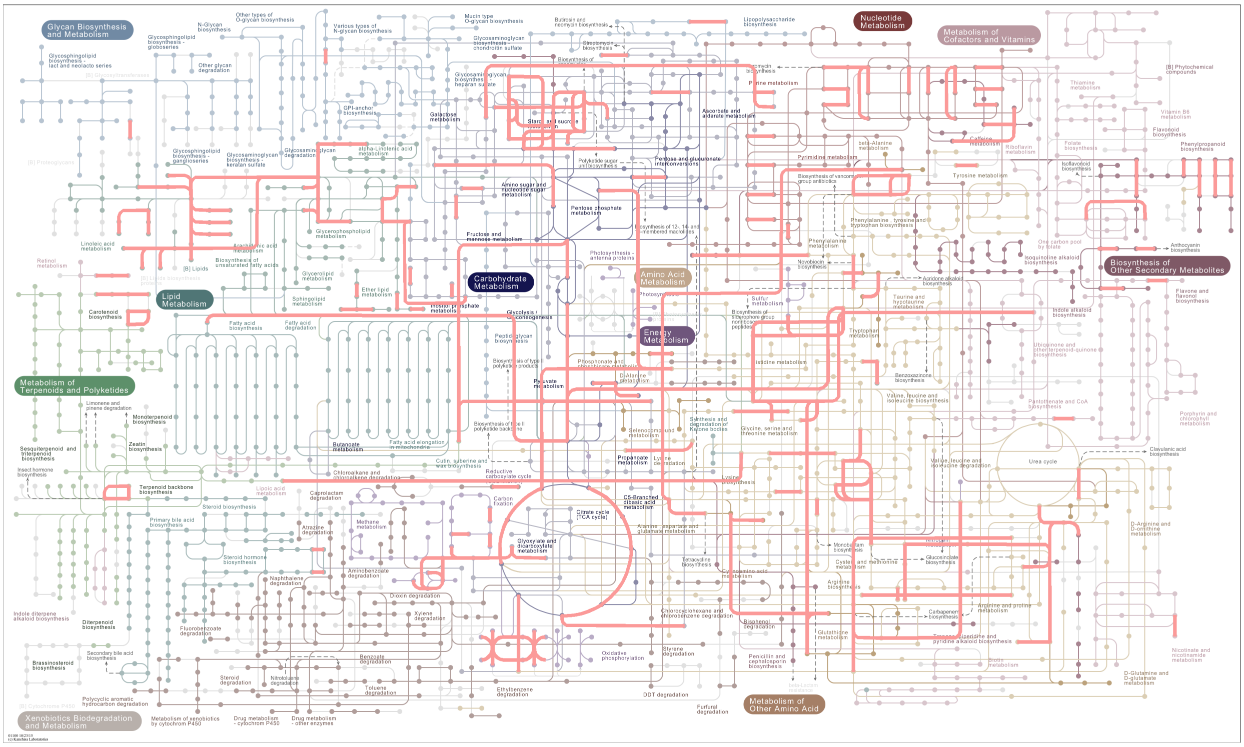

Supplementary Figure 5. Global overview of metabolism. The EC numbers characterized for *S. tuberosum* (potato) were retrieved from the BRENDA database and overlayed in broad light rose on top of a desaturated version of the KEGG metabolic map 01100. The overlaying of data in this manner gives a birds-eye view of which parts of metabolism have been studied in greater detail.
